# Multivariate analysis of the effect of staining beverages on the optical properties of two provisional restorative materials

**DOI:** 10.4317/jced.60431

**Published:** 2023-07-01

**Authors:** Marco Sánchez-Tito, Daniel Blanco-Victorio, José Chauca-Carhuajulca

**Affiliations:** 1 Faculty of Sciences and Philosophy, Universidad Peruana Cayetano Heredia, Lima, Peru; 2 Faculty of Health Sciences, Universidad Privada de Tacna, Tacna, Peru

## Abstract

**Background:**

To evaluate the effect of staining beverages on the color stability, translucency and gloss of two provisional restorative materials.

**Material and Methods:**

Sixty discs (8 mm x 2 mm) were manufactured for Duralay and Protemp 4. The discs were randomly divided according to the beverages: tea, coffee, wine, Coca-Cola and “Chicha morada” (n=12). The discs were polished and initial recordings of color and translucency were made with a spectrophotometer, and the gloss was measured with a glossmeter. The discs were immersed for 5 days in each of the beverages at 37◦C, and the color, translucency and gloss were recorded again. The differences between the initial and final records were calculated to obtain the values of ΔE, ΔTP, ΔGU. For the analysis, the two-way MANOVA model was chosen, and the significance level was set at 5%.

**Results:**

A significant interaction was observed between the type of material and the staining beverages on the changes observed in the values of ΔE, ΔTP, ΔGU (*p*<0.05). ΔE values for Duralay and Protemp 4 were affected by coffee (7.48±1.53) and wine (11.02±1.07), respectively. The greatest change in ΔTP for Duralay were generated by tea (-1.79±0.62), and coffee (-5.65±0.66) for Protemp 4. Gloss was affected mainly by coffee for both materials (Duralay = -6.44±1.17 , Protemp 4 = -8.28±1.09).

**Conclusions:**

The type of material and the pigment drinks act together to influence changes in color, translucency and gloss. The methacrylate-based resin was more stable than the bis-acrylic resin to changes in color, translucency and gloss.

** Key words:**Color, translucency, gloss, staining solutions, interim restorations.

## Introduction

Provisional restorations play an important role in the success of restorative treatment in dentistry (1). Provisional restorations restore the aesthetics, biological, and mechanical functions such as the restoration of occlusion, resistance to wear, precision in the marginal adaptation and optical properties during different periods of time until the change for definitive restorations (2).

Provisional restorations must meet aesthetic characteristics in shape, color, gloss and translucency that approximate them to the definitive restorations, and consequently to the natural teeth (3). Failure to comply with these characteristics can impair the aesthetic appearance of the restorations (1,3).

There are various materials that are used to manufacture provisional restorations, including self-curing, photo-curing and thermo-curing resins (4). Polymethyl methacrylate (PMMA) presents a high resistance, achieving accepTable aesthetic levels and allowing temporary restoration of function (5). However, it has an exothermic release during its polymerization that is accompanied by shrinkage and poor marginal sealing (1). Bis-acrylic resins present a low exothermic reaction, low shrinkage level and better marginal adaptation, improvement in surface micro-hardness, while presenting quite acceptable color stability over long periods of time (6).

The aesthetic properties of provisional restorations cannot be predicted solely based on the chemical composition of the material, factors such as surface finish can contribute to the properties of resisting pigmentation, considering that a porous surface exhibits a greater possibility of staining than a material properly polished (7).

Discoloration of provisional restorations can be caused by the consumption of various foods and beverages (8). On the market, there are products with the ability to generate pigmentation or color change in provisional restorations, including industrialized beverages such as coffee, carbonated beverages, processed juices, tea, wine, fruit juices, among others (9-12).

Janani *et al*., demonstrated that provisional materials and staining solutions act together and are significant factors that affect color stability. Furthermore methacrylate-based resins were more sTable to color change than bis-acrylic resin (9). Mazaro *et al*., observed that acrylic resins were more sTable to color change than bis-acrylic resins, while coffee was the beverage with the greatest staining potential (10). Kotnarin *et al*., also reported that bis-acrylic resins showed greater color change than methacrylate-based resins (11).

Despite the existence of reports on the color change of different provisional restoration materials using various staining solutions, the literature continues to be limited to the study of other properties such translucency and gloss. The aim of this study was to evaluate the effect of staining beverages on the color stability, translucency and gloss of two provisional restorative materials. The null hypothesis tested in this study was that there is no significant interaction effect between the type of restorative material and staining beverages on color, translucency, and gloss.

## Material and Methods

-Sample size calculation 

The sample size was calculated using the Real Statistics resource Pack software (Release 7.6). A power of 0.9, and a partial ɳ2 effect size of 0.06 for the interaction between the two factors were considered. Resulting in a total of 120 samples.

-Specimen preparation

In this study, an autopolymerizing PMMA resin and a bis-acrylic resin were used. Details of the restorative materials are presented in Table 1. The discs were prepared in a metal mold with a diameter of 8 mm and a height of 2 mm (11). Each material was prepared according to the manufacturers’ instructions. Materials were inserted in a single increment into the molds. An acetate strip and a glass plate were placed on the mold to promote removal of excess material and ensure a flat, parallel surface for easy reading of the samples (11). The bis-acrylic resin samples were rubbed for 20 s with a gauze soaked in alcohol to remove the inhibiting layer (10). The samples were visually inspected to discard those with bubbles or surface alterations.

**Table 1:**
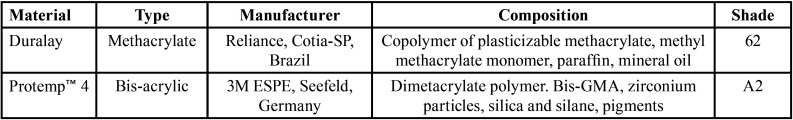
Provisional materials used in this study.

To standardize the surface of the samples, silicone sanding discs (3M ESPE) were used in the sequence of P240, P320 and P360 for 20 s under refrigeration (7). The samples were polished with a goat hair brush (Becht, Labordental Ltda, Sao Paulo, SP, Brazil) and extra fine grain polishing paste (2-4 microns, Diamond Excel, Dentscare, Ltda., Joinville-SC, Brazil ) using a micromotor under a rotation of 18,000 rpm for 1 min. The samples were washed with distilled water for 30 s and placed in an ultrasonic bath for 10 min (7). Finally, The samples were stored in distilled water at 37°C for 24 h until use (12).

-baseline measurement of the parameters

Initial color measure (T1) was performed using the CIE L*a*b* coordinate criteria (13), with a VITA Easyshade® spectrophotometer (VITA Zahnfabriik, Bad Sackingen, Germany). The color was taken on a light gray background to avoid contrast and to be able to standardize the process. The tip of the spectrophotometer was located perpendicular to the center of the disk (12). Measurements were performed in triplicate and the value was averaged for recording.

Translucency was measured using the CIE L* a* b* parameter against a black and white background using a VITA Easyshade® spectrophotometer. The tip of the spectrophotometer was positioned perpendicular to the center of the disk. The Translucency (TP1) was calculated using the following formula: TP = [(L*b - L*w)2 + (a*b – a*w)2 + (b*b – b*w)2]1/2 (14), where L*w, a*w and b*w belong to the white background and L*b, a*b and b*b belong to the black background.

Initial gloss (GU1) was measured with a BEVS1506 glossmeter (BEVS Industrial Co., Ltd. Guangzhou, Guangdong, China), with a measurement area of 2mm x 2mm and a light incidence geometry of 60° (15). The values obtained were expressed in gloss units (GU). A plastic jig was used to block and eliminate ambient light interference and allow reproducibility of measurements that were performed in triplicate for each sample.

-Grouping of the samples

The samples were randomly distributed into 12 groups (n = 12) using the Research Randomizer Form 4.0 software (Social Psychology Network, Middletown, CT, USA). The samples were placed individually in a container, 10 mL of the beverages were added, and the containers were stored at 37°C for 5 days. The solutions were changed every 24 h. For the coffee groups (Nescafé tradición, Nescafé®, Nestlé Brasil Ltda., Araras, SP, Brazil), 4 g of coffee were dissolved in 300 ml of boiling distilled water (11). After 10 min the solution was filtered through filter paper number 1. For the black tea group (Lipton, Unilever Korea Co., Ltd., Seoul, Korea) four tea bags were immersed in 300 ml of boiling distilled water, the solution was filtered through filter paper number 1. (9). For the wine groups, a Carménère wine was used (Chamán Gran Reserva, Viña Santa Cruz, Colchagua, Chile). For the Cola groups, Coca-Cola© (The Coca-Cola Company, Lima, Peru) was used. For the Chicha groups, a commercial purple corn-based beverage “Chicha morada” (Leche Gloria S.A. Lima, Peru) was used, Distilled water was used as a control group.

-Recording parameter differences

After the immersion period in the beverages, the parameters were measured again. Color differences (ΔE) were calculated considering the initial values of L, a and b (T1) and the final values after the immersion time (T2), using the following formula: ΔE = [(ΔL*)2 + (Δa*)2 + (Δb*)2] ½. The differences in translucency were calculated from the difference between the calculated values: ΔTP = PT2 – PT1 (16). The difference in gloss was calculated from the following formula: ΔGU = GU2 – GU1 (15).

-Statistical analysis

Data was analyzed using Stata® 17 Software (StataCorp LP, College Station, TX, USA), As there are two independent grouping variables and three continuous dependent variables, the two-way MANOVA model was chosen to test whether the non-metric values of the independent variables determine the equality of vector means of a series of groups on the dependent variables. To visualize the variation of multivariate null hypotheses (H) relative to error covariation (E), a HE plot was processed with the Rstudio software (version 3.6.1; R Foundation for Statistical Computing, Vienna, Austria). The significance level was set at 5%.

## Results

Data from the control groups were not included in the analysis, as they were considered as outliers, and the differences between the measurements before and after immersion in the staining beverages were not significant. Two-way MANOVA results showed that there is a significant interaction between the type of Material and the Staining beverage (Table 2), because the *p* value is very small for the multivariate test. This finding indicates that factors act together to influence color change, translucency, and gloss parameters. The effect size (ɳ2p = 0.458) indicates that the variability detected in the parameters is explained in 45.8% due to the interaction between the type of material and the staining beverages.

**Table 2:**
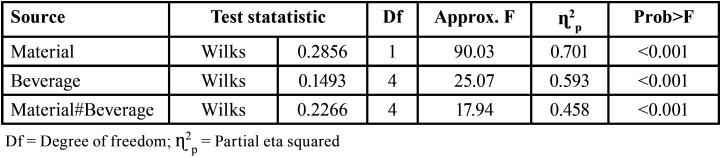
MANOVA results for main effects and interaction.

**Figure 1:**
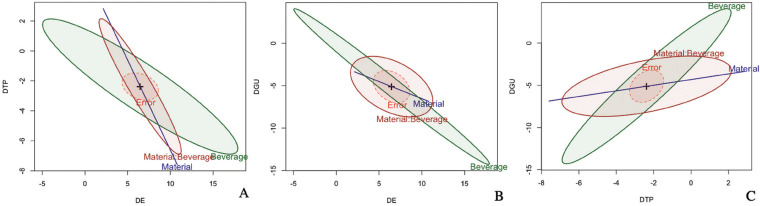
HE plots for the two-way MANOVA scaled for significance of effect according to the factors type of material, beverage, and their interaction. (A). HE plot for effects on ΔE y ΔTP. (B) HE plot for effects on ΔE y vGU. (C) HE plot for effects on ΔTP y ΔGU.

The HE plot showed the size and orientation of hypotheses variation in relation to error as ellipsoids (Fig. 1). The HE plots showed that the multivariate test is significant (*p*<0.05). Each panel shows the bivariate ellipsoids in terms of the variables material, beverage, and their interaction. With all the combinations in the HE plots, we can observe that the ellipsoids of the interaction and of the main effects are highly significant since they are projected outside the ellipsoid of the error. In more detail, for the ΔE*ΔTP y ΔE*ΔGU cases, the significant Material:Beverage measurement interaction occurs primarily in terms of between-group differences in the ΔE response, with little contribution from ΔTP. In the case of ΔTP*ΔGU, the significant interaction is mainly due to the differences between the groups in the ΔTP response.

**Figure 2:**
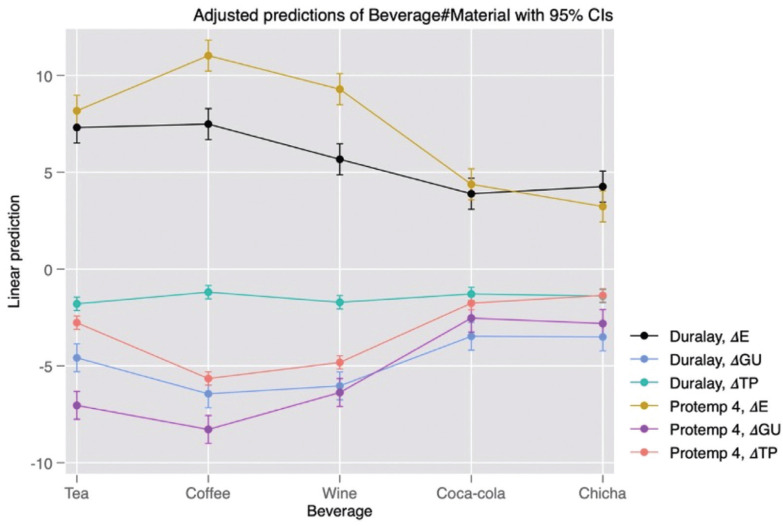
Adjusted predictions from the margins for beverages and type of provisional restorative material.

Figure 2 shows the behavior of the combined data of the type of material and beverage on the optical parameters. In the case of ΔE, it can be observed that there were differences between the samples of both materials when they were subjected to coffee and wine. For ΔTP, the differences between the materials were observed when the samples were subjected to tea, coffee and wine. Finally, in the case of ΔGU, the differences were observed when tea and coffee were used as staining solution. The comparisons of pairs were made through the linear prediction of the margins, the results are shown in Table 3.

**Table 3:**
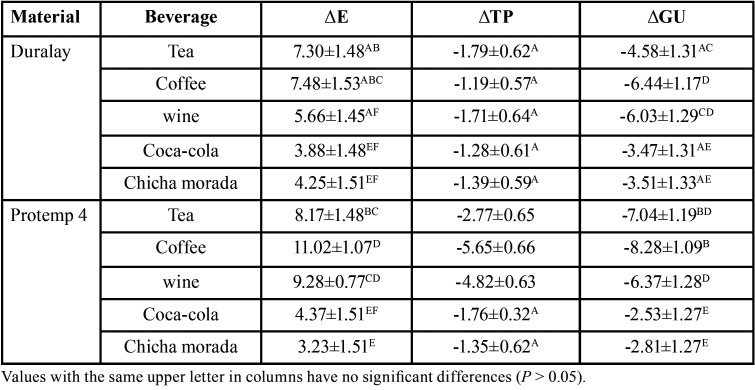
Mean and standard deviation of differences in color, translucency and gloss by type of provisional restorative material and beverages.

## Discussion

Although provisional restorations are intended to be used for a limited period of time, aesthetic properties are an important aspect, especially when the treatment reaches areas with high aesthetic requirements such as the anterior region (1,3,16). Among the aesthetic properties that must be considered in these materials, we have color stability, translucency and gloss, among others (1). It is known that these properties can be affected by various factors, including the consumption of food and beverages, which have the potential to penetrate the matrix of the material, behaving as extrinsic pigmentation (3,9-12).

Several studies have evaluated the color stability of various restorative materials, including different evaluation periods. It is expected that the longer the immersion time, the greater the color change. However, these prolonged periods of immersion are not comparable to the time that the materials would be in contact with the staining solutions under clinical conditions. Therefore, the results must be interpreted carefully. In this study, to simulate the effect of one year of drinking beverages, it was considered that the average consumption time of a 250 ml cup of coffee or tea is approximately 20 min, with a consumption of two cups per day (17). An immersion time of 120 hours or 5 days was estimated.

In general, previous studies individually evaluate the effect of staining agents on the aesthetic properties of various temporary prosthetic restoration materials, without considering other properties and factors that may influence their behavior. In this study, it was considered to establish a multivariate model to verify if the interaction between the type of material and the type of staining beverage have an impact on aesthetic properties such as color, translucency and gloss. The results indicate that the null hypothesis of no significant effect of the interaction of the type of material and staining beverages on color, translucency, and gloss was rejected (Δ = 0.2266, F = 17.94, *p*<0.001, ɳ2p = 0.458). These results show that there is a significant interaction between the type of material and the staining beverages, that is, the factors act together to influence the color, translucency and gloss parameters.

When analyzing the behavior of color, we can observe that the highest values of ∆E were in the Protemp 4 group when tea, coffee and wine were used as staining beverages. In the literature, a value of ∆E ≥ 3.7 is proposed as a threshold to establish a clinically perceptible color difference (18). In this study, all the combinations of materials and staining beverages showed a color change above 3.7, with the exception of the Protemp 4 samples immersed in chicha morada. For both types of materials, coffee had a larger effect on color change. Consequently, both materials showed clinically unacceptable color changes, although Duralay was more sTable to color change. These results are in agreement with those reported by other authors.

Kotnarin *et al*. (11), observed that Protemp 4 discs when subjected to coffee had ∆E values of 5.26±1.33 to 16.69±3.18 at 7 and 16 days, respectively. Costa *et al*. (19), evaluated the color stability of a methacrylate-based resin (Duralay) and a bis-acrylic resin (Protemp 4) subjected to Coca-Cola, wine and coffee. Their results showed that in the case of Duralay the values of ∆E were 1.08±1.12, 0.94±0.84 and 3.92±2.43 for each beverage; while for Protemp 4, the values were 4.71±3.36, 11.73±3.47 and 10.60±1.66, these results showed that the methacrylate-based resin had better color change stability.

The greater color change observed in the bis-acrylic resin may be due to the high diffusion coefficient compared to methacrylate-based resins, so bis-acrylic resins have a higher water absorption, which leads to a greater color change (20). Another factor that can influence the pigmentation of bis-acrylic resins is that they are more polar than PMMA polymers, which generates a greater affinity for water and other liquids (21). Bis-acrylic resins have bis-GMA (bisphenylglycidyl dinthecrylate) as resin matrix, this monomer is hydrophilic, and due to its hydroxyl group it can form hydrogen bonds with water (22). Due to the heterogeneous composition of bis-acrylic resins, it has been observed that after the setting reaction, gaps are formed in their structure, which would facilitate pigmentation (23), (Table 3).

In relation to the staining beverages, they can have different polarities, and this factor can influence the potential to penetrate the matrix of the restorative material or generate a pigmentation just on the surface (24). In this study, high consumption beverages such as tea, coffee, wine, a carbonated drink and a traditional drink made from purple corn were selected. Tea is high in tannins and coffee contains a high amount of chromogens, which are pigments with high molecular weight that have the potential to stain provisional restorations (25). Coffee and tea has the quality of being a low polar solution, which allows it to easily penetrate into the polymeric matrix (26). On the other hand, staining solutions such as wine have high polarity, so their staining potential occurs on the surface of the material.

The cola drink had low staining potential when compared to other beverages such as tea, coffee, and wine. In general, it has been observed that cola drinks have low staining potential in resin materials, despite being more acidic than other drinks. This could be explained by the presence of phosphate ions in the drink, which has been associated with the prevention of damage to the surface of the resin material (27). The purple corn beverage has a high content of anthocyanins that, associated with low pH levels in the drink, can cause damage to the surface of the material and allow the penetration of pigments, demonstrating a high staining potential in resin materials (28). In this study, the color changes produced by the “chicha morada” beverage were less than those observed in the other beverages, this can be explained because a commercial product based on purple corn was used, which could have decreased the staining potential observed in the natural beverage.

The results showed that the translucency was reduced in all samples. These findings are in agreement with other study that have reported a decrease in translucency in methacrylate-based resins (29). As the material matrix absorbs the solution, the pigment constituents penetrate deeper, decreasing light transmittance and translucency. In the literature, a value of ΔTP = 2 has been reported as the threshold of perceptibility (15,17). In this study, the decrease in translucency was greater in the Protemp 4 group compared to Duralay, and was mainly affected when coffee and wine were used as staining solutions, thus the changes in decreased translucency were clinically perceptible.

Changes in gloss are clinically perceptible with variations greater than 6.4 GU (30). When analyzing the gloss values in the groups, it was observed that the Duralay discs were more sTable to the decrease in gloss than those of Protemp. Particularly, the change for Protemp can be considered clinically perceptible when they were immersed in tea and coffee, since the change was greater than the threshold value.

The optical properties of dental materials depend on various factors that cannot be reproduced in a laboratory environment. Factors, such as the presence of saliva, tissues surrounding the restoration, the background of the oral cavity, among others, affect the optical properties and their clinical perceptibility. Thus, further studies that evaluate these characteristics in provisional restorations in situ, could provide a greater understanding of the behavior of provisional restorative materials.

## Conclusions

The results showed that there is a significant interaction between the type of material and the staining beverages, acting together to influence the observed changes in color, translucency and gloss. The methacrylate-based resin was more stable than the bis-acrylic resin to changes in the dependent variables. Coffee, tea, and wine were the beverages that negatively affected color, translucency, and gloss, promoting values higher than those clinically acceptable.

## References

[1] Chiramana S, J Dev RR, Banka M, Pssv Sirisha, Rao K, Chvn SK (2019). Provisional Restoration in Prosthodontics : A Review. J Adv Med Dent Scie Res.

[2] Regish KM, Sharma D, Prithviraj DR (2011). Techniques of fabrication of provisional restoration: an overview. Int J Dent.

[3] Christensen GJ (1996). Provisional restorations for fixed prosthodontics. J Am Dent Assoc.

[4] Patras M, Naka O, Doukoudakis S, Pissiotis A (2012). Management of provisional restorations' deficiencies: a literature review. J Esthet Restor Dent.

[5] Prasad DK, Shetty M, Alva H, Prasad DA (2012). Provisional restorations in prosthodontic rehabilitation - concepts, materials and techiniques. NUJHS.

[6] Bharadwaj K, Salagundi BS, Regish KM (2017). Provisional restorations in crown and bridge. International Journal of Current research.

[7] Tupinambá ÍVM, Giampá PCC, Rocha IAR, Lima EMCX (2018). Effect of different polishing methods on surface roughness of provisional prosthetic materials. J Indian Prosthodont Soc.

[8] Prasad DK, Alva H, Shetty M (2014). Evaluation of colour stability of provisional restorative materials exposed to different mouth rinses at varying time intervals: an in vitro study. J Indian Prosthodont Soc.

[9] Janani S, Reddy PM, Tr R, Gupta B (2016). Comparative evaluation of color stability of four provisional restorative materials : An invitro study. IP Ann Prosthodont Rest Dent.

[10] Mazaro JVQ, Minani LM, Zavanelli AC, Mello CC de, Lemos CAA (2015). Evaluation of color stability of different temporary restorative materials. Rev Odontol. UNESP.

[11] Kotnarin N, Nagaviroj N, Kanchanavasita W (2018). The effect of staining solutions on the color stability of the provisional restorative materials. M Dent J.

[12] Tekçe N, Tuncer S, Demirci M, Serim ME, Baydemir C (2015). The effect of different drinks on the color stability of different restorative materials after one month. Restor Dent Endod.

[13] Joiner A (2004). Tooth colour: a review of the literature. J Dent.

[14] Lee YK (2016). Criteria for clinical translucency evaluation of direct esthetic restorative materials. Restor Dent Endod.

[15] Rocha RS, Ruano V, de Souza MY, Salomao FM, Bresciani E (2022). Color and surface gloss stability of bis-acryl and resin composite after exposure to cigarette smoke. Braz Dent Sci.

[16] Kim JE, Choi WH, Lee D, Shin Y, Park SH, Roh BD (2021). Color and Translucency Stability of Three-Dimensional Printable Dental Materials for Crown and Bridge Restorations. Materials (Basel).

[17] Czarniecka-Skubina E, Pielak M, Sałek P, Korzeniowska-Ginter R, Owczarek T (2021). Int J Environ Res Public Health. Consumer Choices and Habits Related to Coffee Consumption by Poles.

[18] Johnston WM, Kao EC (1989). Assessment of appearance match by visual observation and clinical colorimetry. J Dent Res.

[19] Costa IA da F, Lima EMCX (2018). Effect of Colorant Solutions on the Color Stability of Provisional Prosthetic Materials. Brazilian Journal of Oral Sciences.

[20] Yannikakis SA, Zissis AJ, Polyzois GL, Caroni C (1998). Color stability of provisional resin restorative materials. J Prosthet Dent.

[21] Haselton DR, Diaz- Arnald AM, Dawson DV (2005). Color stability of provisional crown and fixed partial denture resins. J Prosthet Dent.

[22] Kerby RE, Knobloch LA, Sharples S, Peregrina A (2013). Mechanical properties of urethane and bis-acryl interim resin materials. J Prosthet Dent.

[23] Bitencourt SB, Kanda RY, Freitas Jorge C, Barão VAR, Sukotjo C, Wee AG (2020). Long-term stainability of interim prosthetic materials in acidic/staining solutions. J Esthet Restor Dent.

[24] Borges AL, Costa AK, Saavedra GS, Komori PC, Borges AB, Rode SM (2011). Color stability of composites: effect of immersion media. Acta Odontol Latinoam.

[25] Bravo L (1998). Poly-phenols: chemistry, dietary sources, metabolism, and nutritional significance. Nutr Rev.

[26] Eldwakhly E, Ahmed DRM, Soliman M, Abbas MM, Badrawy W (2019). Color and translucency stability of novel restorative CAD/CAM materials. Dent Med Probl.

[27] Aliping-McKenzie M, Linden RW, Nicholson JW (2004). The effect of Coca-Cola and fruit juices on the surface hardness of glass-ionomers and 'compomers. ' J Oral Rehabil.

[28] Acuña ED, Delgado-Cotrina L, Rumiche FA, Tay LY (2016). Effect of the purple corn beverage "Chicha morada" on composite resin during dental bleaching. Scientifica.

[29] Elsaka S, Taibah S, Elnaghy A (2020). Effect of staining beverages and bleaching on optical properties of a CAD/CAM nanohybrid and nanoceramic restorative material. BCM Oral Health.

[30] Rocha RS, Fagundes TC, Caneppele T, Bresciani E (2020). Perceptibility and acceptability of surface gloss variations in dentistry. Oper Dent.

